# The impact of the COVID-19 pandemic and related measures on people with psychiatric disorders in a small town in Greece

**DOI:** 10.1192/j.eurpsy.2023.497

**Published:** 2023-07-19

**Authors:** S. Kostarelou, K. Argyropoulos, V. Mproumas, D. Avramidis, K. Assimakopoulos, P. Gourzis, E. Jelastopulu

**Affiliations:** ^1^MSc Public Health; ^2^Public Health, School of Medicine, University of Patras, Greece, Patras; ^3^Psychiatry, Messolonghi General Hospital, Messolonghi; ^4^School of Medicine, Univesrity of Patras, Greece; ^5^Psychiatry, School of Medicine, University of Patras, Patras, Greece

## Abstract

**Introduction:**

A pandemic can have significant effects on people’s emotional wellbeing. Infection control measures such as social distancing can lead people to feel isolated and to increased feelings of fear, anxiety, anger, and sadness. Recent research showed a worrying increase in depression and anxiety disorders, general distress, and sleep disorders. People who already suffer from a mental illness may be more vulnerable to stress caused by the pandemic and may experience a deterioration of already preexisting symptoms of anxiety and depression.

**Objectives:**

The purpose of the present study was to assess the pandemic’s psychological impact on people with preexisting mental illness, to investigate their COVID-19-related fear, anxiety, and depression in association with various variables and to explore their behavioral responses regarding the measures against the pandemic.

**Methods:**

A cross-sectional study was conducted from March to May 2022 in the outpatient mental health clinic of a provincial hospital in Greece. Participants were patients, who were not fully disorganized and have been diagnosed with a mental illness before the COVID-19 pandemic. Among the 50 adult patients, 11 lived in assisted living facilities. The study included sociodemographic questions, questions about fear of COVID-19, negative feelings, safety measures and behaviors, disease progression, and compliance with their therapists. DASS-21 scale was used to measure the 3 subscales of emotional states.

**Results:**

Sadness was reported as the most unpleasant emotion of the lockdown, following by hopelessness and denial. Several participants (36%) reported high levels of COVID-19-related fear, mainly patients living in the community and not in assisted facilities (40.1% vs 18.2%) and males compared to females (42.1% vs 32.3%). The majority (70%) declared high compliance with the therapy. A moderate to severe deterioration in disease progression during the pandemic was observed in 28%, mainly in females compared to males (38.7% vs 10.6%). Based on DASS-21 the mean scores ranged from moderate to severe symptoms in depression (16.2/42), anxiety (14.3/42), and stress (18.4/42) without a statistically significant correlation with age, gender, and living situation. However, 34%, 22% and 56% screened positive for severe and extreme severe depression, anxiety, and stress, respectively.

**Conclusions:**

The study revealed a substantial proportion of patients with mental disorders to experience unpleasant emotions and increased levels of psychological distress and highlights the need for supportive mental health services to address the increased mental health symptoms in people with pre-existing mental illnesses during a pandemic.

**Disclosure of Interest:**

None Declared

